# Exploring the critical waste factors affecting highway construction projects in Pakistan

**DOI:** 10.1371/journal.pone.0323841

**Published:** 2025-05-28

**Authors:** Usman Aftab, Farrokh Jaleel, Mughees Aslam, Javed Ahmed Khan Tipu

**Affiliations:** 1 International Islamic University Islamabad (IIUI), Department of Mechanical Engineering/ Engineering Management, Pakistan; 2 NUST Balochistan Campus, National University of Science and Technology (NUST), Quetta, Balochistan, Pakistan; 3 International Islamic University Islamabad (IIUI), Department of Mechanical Engineering/ Engineering Management, Pakistan; Shandong University of Technology, CHINA

## Abstract

Highway construction projects are known for their propensity to consume enormous quantities of materials and their susceptibility to generate considerable waste. Adequate research has been conducted on identifying the causes of waste produced in building projects, however, there are limited studies available on identifying causative factors for highway projects. This study aims to identify and evaluate the causes and factors of waste generated on highway projects using a literature review and questionnaire survey technique. Causes leading to waste in highway projects were identified from the literature as well as from highway construction experts. Subsequently, quantitative data were collected from 127 highway construction professionals using a Likert Scale questionnaire survey, which was ranked using the Relative Importance Index (RII) and further analyzed by using a very robust Factor Analysis (FA) technique. RII results highlight the most significant causes of waste in highway construction, while FA suggests the main factors contributing to the waste in highway projects. The top five most significant causes of waste revealed by this study were: (1) mistakes of surveyors, (2) faulty drawings, (3) incompetence of quantity surveyors, (4) faulty/substandard work, and (5) poor workers’ skills, whereas the seven waste factor groups evaluated by the study were: (1) design, (2) storage, (3) survey, (4) workers, (5) waste management, (6) site management, and (7) external. This study further suggested waste management and mitigation strategies for highway construction projects corresponding to each factor group. This is a novel study on waste generation in highway projects in Pakistan and will assist academia and industry practitioners in understanding and controlling construction waste generation in highway projects during various stages of project execution.

## 1. Introduction

Highway construction is distinct from other types of construction projects. Its linear and elongated nature, the propensity to consume enormous quantities of construction materials, and susceptibility to waste generation pose unique management and logistical challenges to the project team and other stakeholders [1,2]. Materials constitute more than 50 percent of the overall project cost in highway construction projects, signifying the need for proper management and optimal utilization [3].

Studies have highlighted substantial material wastage in highway construction projects. In the USA, cumulative material wastage has been estimated at up to 29.4% across different stages of the project lifecycle [4]. In Indonesia, researchers found aggregate wastage at 26% and concrete wastage at 5.3% in highway projects [3]. Similarly, a study in the UK reported overconsumption of concrete (10.9%), tarmac (22%), and cement (79.7%) in road maintenance projects [5]. Given the significant material usage in highway construction, even minor reductions in waste can lead to substantial financial savings [1].

Globally, waste management strategies in construction projects have focused on waste reduction, reuse, and recycling instead of on-site disposal [6,7]. These strategies are typically based on identifying the root causes of waste generation [8,9]. Researchers have extensively examined these causes and categorized them into factor groups such as design, equipment, labor, material handling, procurement, and storage [9–11]. Understanding the relationships between these factors has enabled the development of waste management frameworks and models.

Recent studies have provided valuable insights into construction waste management. In Morocco, researchers identified 31 root causes of waste in building construction projects and grouped them into six clusters: Material; Planning and Coordination; Subcontractors and Workers; People and Financial; Development Strategies; and External Factors [9]. Similarly, a study on building projects analyzed 28 waste causes and proposed preventive strategies for the seven most significant ones [12]. A study in Vietnam examined 19 waste causes and their impact on project cost performance [13].

Despite these advancements, research on material waste in infrastructure and highway construction remains limited. A study in Qatar assessed 26 generic causes of waste in infrastructure projects and categorized them into six clusters: Design, Logistics, Procurement, Execution, Design, and Others, using Structural Equation Modeling [14]. In Sri Lanka, researchers focused on a single material—Aggregate Base Course (ABC)—in highway construction, identifying 23 causes of its wastage (19 derived from literature and four from expert input) to develop a waste management framework [15]. However, most existing studies are either generic or concentrated on building construction, with minimal focus on infrastructure projects like highways [14,16]. Furthermore, contemporary research often examines macro-level causative factors while neglecting micro-level causes specific to diverse construction projects. This gap highlights the need for a detailed micro-level analysis of waste causation in highway construction.

To the best of the authors’ knowledge, this is the first study on construction waste management (CWM) in highway projects within the Pakistani context. The study aims to:

1) Identify critical causative factors contributing to waste generation in highway construction projects.
2) Examine the impact of identified factors on material wastage.

## 2. Literature review

### 2.1. Construction Waste (CW) and causes

CW includes materials that are required to be either transported away from the project site or used for a purpose other than specified because of damage, excess use, non-utilization, or specifications issues, etcetera [17,18]. CW has been described as an inefficiency that causes excessive consumption of materials and other project resources [19,20]. Although most of the definitions of CW available in the literature are from the perspective of building construction, they are also applicable to highway construction. The CW in buildings constitutes tens of materials whereas the highway construction involves few materials but in very large quantities [4]. Identification of significant causes of CW helps in formulating a waste management framework for construction projects. Adequate research is available on a host of waste causes specific to building construction projects, whereas little research is available on waste causes specific to highway and infrastructure projects. Many studies have identified waste causes for the literature and experts/case studies for the questionnaire survey based on the Likert Scale [9,12,13]. Table 1 shows details of studies on the most significant causes of CW reported in the literature. It shows that “frequent design changes” is the most repeated cause in the selected studies, followed by “lack of storage,” “lack of training,” “material quality issues,” and “rework”. In the context of highway projects, the most significant causes of Aggregate Base Course (ABC)waste established by a recent study were “laying ABC without road shoulders”, “using ABC in place of the soil” and “frequent design changes”; whereas the significant consequences of ABC wastage were “cost overruns”, “time overruns” and “contractors financial issues” [15].

**Table 1:** CW Causes in Literature.

Source	Scope	Causes	Factors	Responses	Methodology	Most Significant Waste Causes
[16]	*Roads waste in Indonesia*	18	3	23	Ranking based on Mean	• Lack of equipment • Poor scheduling • Unskilled labour • Frequent design changes • Extreme Weather
[21]	Buildings/ UAE	13	4	56 (46%)	Ranking based on Mean	• Damages during transport • Poor quality of products • Inadequate material • Over-ordering of materials • Poor advice from suppliers
[22]	Buildings/Jordan	25	6	160 (67%)	Ranking based on Frequency, Severity, and Importance Index	• Frequent design changes • Rework • Poor contract documents • Inadequate storage • Lack of waste reduction plan
[23]	Highrise buildings/ Vietnam	19	5	128 (43%)	Ranking based on Mean	• Poor supervision • Poor planning/scheduling • Bureaucracy • Poor supervision • Poor distribution of resources
[24]	Buildings/ Turkey	34	7	66 (100%)	Ranking based on RII	• Frequent design changes • Design errors • Cutting uneconomical shapes • Incorrect material specifications • Lack of supervision
[25]	Buildings/ Pakistan	19	6	38 experts	Ranking based on Mean	• Poor Workers Skills • Poor supervision • Poor management • Equipment malfunction • Lack of waste reduction plan
[26]	Generic/ Global	80	6	–	Review Article	• Frequent design changes • Inadequate storage • Poor handling • Extreme weather • Ordering mistakes
[27]	Buildings/ India	22	–	–	–	• Frequent design changes • Rework • Poor planning/scheduling • Wrong ordering • Excess consumption
[28]	Buildings/ Jordan	38	5	73 (84%)	Ranking based on Mean	• Frequent design changes • Rework • Wrong Specifications • Cutting of materials • Poor site layout
[15]	*Highway Projects/Global*	23		137 (45%)	Ranking based on Mean	• ABC laying without shoulders • ABC use in place of soil • Frequent design changes • Increase internal transport time • Material segregation
[12]	Buildings Bangladesh	28	5	113 (80%)	Ranking based on Relative Importance Index (RII)	• Deposited material in a public place • Inadequate storage • Lack of training • Solid Waste • Absence of guidelines for workers
[29]	Building India	14	6	45	Ranking based on Mean	• Frequent design changes • Poor quality of materials • Poor technology • Extreme weather • Wrong specification
[9]	Generic/ Bangladesh	31	6	330 (82%)	Ranking based on Mean/ FA	• Late payment • Lack of training • Lack of 3Rs • Accidents • Multiple levels of subcontractors
[14]	*Infrastructure/ Qatar*	23	6	167 (33%)	SEM	• Take-off error • Unforeseen incidents damaging site • Design errors • Extreme weather • Lack of design information

The literature identifies a long list of causes of CW, which are subsequently grouped into factors/clusters based on mutual similarity. The main factors evaluated by the researchers include design and contract documents, site management, supervision, procurement, human resources, execution etcetera. Table 2 shows the frequency of each waste factor mentioned in the literature. The most repeated factor groups were design & contract documents and site management/ supervision, followed by execution/operations, procurement, and people/workers, handling storage, and external factors.

**Table 2:** CW Causative Factors in Literature.

Source Main Factors	[9]	[24]	[23]	[10]	[11]	[14]	[30]	[21]	[26]	[28]	[16]	Frequency
Design and contract documentation		✔		✔	✔	✔		✔	✔	✔	✔	8
Procurement		✔		✔		✔		✔				4
Handling		✔					✔			✔		3
Storage		✔			✔		✔					3
Worker/People/ HR		✔	✔	✔						✔		4
Site management and supervision		✔	✔		✔	✔	✔		✔	✔	✔	8
External		✔							✔	✔	✔	4
Material	✔		✔								✔	3
Execution/ Operations			✔		✔	✔		✔			✔	5
Transportation/logistics				✔		✔						2
Material & Equipment						✔			✔			2

### 2.2. Construction Waste Management (CWM)

Waste hierarchy or 3 R (reduction, reuse, and recycling) has been used as a guiding principle for formulating CWM practices for decades [6,31]. The EU has also laid out a comprehensive hierarchy for waste management strategies which includes prevention, reuse, recycling, and disposal. Waste prevention has been accorded the topmost priority out of waste management strategies, while disposal in landfills is the last [32]. However, research regarding the prevention of waste, which primarily examines CW causes is insufficient [33]. Many studies have formulated waste management strategies corresponding to the causative factors of waste generation. Selected CWM strategies reported in the literature are listed in Table 3. In the context of highway projects, a recent study in Sri Lanka proposed a framework for CWM of ABC material [15].

**Table 3:** CWM Strategies.

CWM Strategies	Source
Waste management regulations and supervision	[34], [35]
Waste management system (WMS)	[15], [31,36–38]
Awareness of CM	[34]
Fewer design changes Minimize design revision/ late variations	[34], [35]
Research and development in Waste management.	[34]
Training/Human Resource Development in WM	[21], [34,35]
Material usage and storage system	[9], [15,21]
On-site C& CW supervision system Auditing tool	[31], [34]
Environmental management system/ Awareness of climate change	[34], [35]
On-site C&D waste sorting	[31], [34]
Behavior and attitude/Culture	[31], [35,39]
CW quantification and source evaluation.	[9], [31]
Procurement waste minimization strategies.	[31], [39]
Design waste minimization/ Design for flexibility and adaptability	[31], [35]
Legislation on WM	[31]
Comparative waste management studies/ Feasibility study/ waste scenario plan	[11], [31]
Building Information Modeling (BIM) Aided WM	[31], [40]
Off-Site Construction/Prefab/Modular Design	[31], [35]
Administration of 5 M (material, money, machines, and management	[15]

### 2.3. CW in highway projects

CW is a generic term encompassing material waste in all kinds of construction projects, including buildings, infrastructure, highways, etcetera [17,18]. The term Highway CW is specifically used for the materials consumed and wasted in road/highway projects. These materials include Subgrade, Subbase, Base, and Asphaltic materials for the road pavement while concrete and steel for the culvert/bridge structures [3,4]. Building construction projects involve the consumption of a wide variety of materials, including sand, cement, steel, glass, plastics, tiles, stones, paint, etcetera. In contrast, highways/roads use only a few types of materials however their consumption is in enormous quantities with immense susceptibility to wastage [4]. In 2006, a study conducted in the USA quantified wastage rates of gravel, asphalt, crushed stone, and sand for a 1 km road section and estimated wastage of 29.4% during the life cycle assessment of the project [4]. Due to the excessive consumption of aggregate in highway projects, studies have focused on the wastage of aggregate material used in highway projects [1,15,41]. In 2010 in the UK a study on road maintenance projects computed waste percentages/overconsumption of tarmac (22%), cement (79%), and concrete (10%) [5]. Causes of waste generation in highway projects have also been evaluated by a few researchers (See Table 1 above) [15,16,33,41]. Generic causes of waste generation in highway construction were identified through an exploratory study conducted in Indonesia based on a questionnaire survey responded by only a few professionals [16].

### 2.4. Gaps in literature

The bulk of CWM research has been focused on building projects, ignoring waste reduction in highway and infrastructure projects, notwithstanding the impact of huge material wastage on project performance and sustainability [14,16,42]. Moreover, CW prevention is considered the most cost-effective waste management strategy by researchers, still, research on waste prevention is insufficient [33]. The scarcity of research on the prevention of waste in highway and infrastructure projects underlines the gap in existing knowledge.

## 3. Research methodology

**Fig 1:**
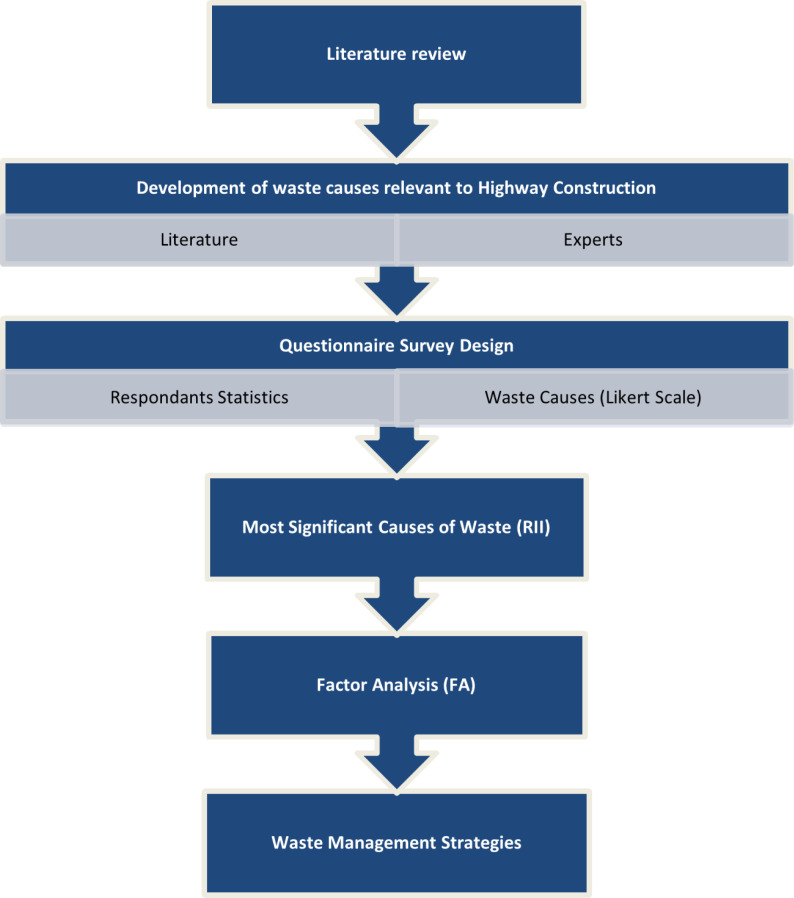
Research Methodology Flow Chart.

This exploratory study follows a mixed research methodology approach. Fig 1 shows the flow chart of the research methodology of this paper. The study starts with a literature review to provide an in-depth understanding of the genesis of waste in construction projects. Thereafter, critical waste causes relevant to the construction of highway projects were developed from the literature review and expert panel input for subsequent assessment through a Likert Scale questionnaire survey. Questionnaire surveys have been extensively used in similar studies by researchers [8,9]. Relative Importance Index (RII) and Factor Analysis (FA) were used for the evaluation of data. Finally, the results were analyzed to recommend waste management strategies corresponding to the evaluated factors contributing to CW in highway projects.

To maintain confidentiality and ensure respondents’ willingness to participate, participation in the survey was entirely voluntary. Written informed consent was obtained from all participants through a built-in consent section within the questionnaire. The questionnaire explicitly stated that by proceeding with the survey, participants acknowledged their informed consent to provide input. Since all responses were s1ubmitted anonymously, it is not possible to re-contact the respondents, except for those who explicitly agreed to a follow-up session, if required. The Institutional Ethical Review Committee (IIUI) has approved the study for further analysis and confirmed that it poses no risk to the participants.

### 3.1. Causes identification

Given the limited research on construction waste (CW) causes in Pakistan, relevant CW causes applicable to highway construction were first identified from global literature. Recognizing that engineering practices and waste generation factors vary across countries, the initial identified causes are tailored to the Pakistani context. This was achieved through expert panel consultations, ensuring that only the most relevant causes were selected for further investigation. The questionnaire survey was designed based on these refined causes, allowing the capture of Pakistan-specific insights while leveraging global research as a foundational reference. Table 1 presents a summary of the details from selected studies conducted in the UAE, India, Pakistan, Indonesia, Jordan, Bangladesh, and Qatar [21–26]. Interestingly, a substantial similarity can be observed in the top significant causes identified in research work conducted in different parts of the world with varying methodologies. Conversely, few studies conducted in the same countries have yielded varied results [27,29]. However, it can be observed that in almost all the studies mentioned in Table 1, initially, waste causes have been adapted from the global literature and later tailored to accommodate local conditions by expert advice, and the same approach was followed in this study as well.

In this study, assistance was sought from an expert panel comprising four project managers (PM) with more than 15 years of experience in highway construction and a master’s degree in construction management. Other similar studies have also included causative factors on the advice of subject experts [15]. A total of 45 causes were shortlisted for this study, out of which 31 were taken from the literature while the expert panel suggested the remaining 14. Table 4 shows the list of HWC included in the questionnaire.

**Table 4:** HWCs included in Questionnaire Survey.

ID	Cause	Source	Knowledge Area
HWC1	Complex Design	Literature	Planning & Design related
HWC2	Frequent Changes	Literature	
HWC3	Faulty Drawings	Literature	
HWC4	Delay in Drawings Distribution	Expert Panel	
HWC5	Less Planning Time	Literature	
HWC6	Taking Off Errors	Literature	Procurement related
HWC7	Ordering Errors	Literature	
HWC8	Suppliers Errors	Literature	
HWC9	Poor Quality and Wrong Specifications	Literature	
HWC10	Delay in Delivery of Materials from suppliers	Literature	
HWC11	Bulk Procurement & delivery in Advance	Expert panel	
HWC12	Lack of Storage Space	Literature	Storage related
HWC13	Multiple Storage Spaces spread along long stretch	Expert panel	
HWC14	Distance of Work Site from storage places	Expert panel	
HWC15	Inappropriate/Inadequate Storage Arrangements	Literature	
HWC16	Mishandling by Equipment during Transportation	Literature	Handing & Equipment related
HWC17	Mishandling of Material during Loading/Unloading	Expert panel	
HWC18	Non-availability of Appropriate Equipment	Expert panel	
HWC19	Non-availability of Appropriate Surveying Equipment	Expert panel	
HWC20	Faulty/Malfunctioning of Equipment	Literature	
HWC21	Poor Workers Skills	Literature	Workers related
HWC22	Poor Capacity of Designers	Literature	
HWC23	Incompetence of Quantity Surveyors (QSs)	Expert panel	
HWC24	Mistakes of Surveyors	Expert Panel	
HWC25	Fast Pace of Work	Literature	
HWC26	Problems with the Attitude and Behaviour of Workers	Literature	
HWC27	Lack of Awareness of Wastage	Literature	
HWC28	Poor Working Conditions for workers	Literature	
HWC29	Lower than the Designed Level of the Subgrade Layer	Expert Panel	Management Related
HWC30	Poor Supervision of Work	Literature	
HWC31	Prolonged Halting of Work by Consultant/ Client	Literature	
HWC32	Halts due to Engineering Practices	Expert Panel	
HWC33	Lack of Coordination among stakeholders	Literature	
HWC34	Absence of Waste Management Plan	Literature	
HWC35	Use of Wrong Construction Methods	Literature	
HWC36	Frequent Movement of Materials from one site to another/ Double-handling	Literature	
HWC37	Material Segregation and Sorting issues	Literature	
HWC38	Faulty/Substandard Work (Requiring Rework)	Literature	
HWC39	Unsuitable site	Literature	Site related
HWC40	Site Restricting Equipment Operation	Expert Panel	
HWC41	Remote Site/ Wilderness (accessibility issues)	Expert Panel	
HWC42	Site Spread over Very Long Length impacts	Expert Panel	
HWC43	Theft and Vandalism incidents	Literature	External
HWC44	Occurrence of Accidents	Literature	
HWC45	Bad Weather Conditions	Literature	

The study comprehensively considers the generation of Highway Construction Waste (HCW) across different stages of life cycle of a highway project, encompassing planning, design, procurement, execution, and site management. The identified HCW causes (HWCs) span these phases, ensuring a holistic assessment of waste generation factors. To evaluate HCW from a life cycle perspective, the study categorizes waste causes based on their origin within different project stages:

a **Planning & Design Phase:** Issues such as complex designs, frequent changes, faulty drawings, and insufficient planning time contribute to waste by necessitating rework and material overuse.
b **Procurement & Storage:** Ordering errors, supplier mistakes, poor material specifications, and inadequate storage arrangements lead to material wastage before usage.
c **Execution & On-Site Handling**: Mishandling of materials during transportation, lack of skilled workers, inappropriate equipment, and poor site conditions contribute to inefficiencies and rework.
d **Management & Supervision:** Lack of coordination, improper supervision, absence of waste management strategies, and halts in work exacerbate waste accumulation.
e **External & Environmental Factors:** Remote site conditions, theft, vandalism, and accidents add further layers to HCW generation.

### 3.2. Questionnaire development

The questionnaire was structured in two sections: Section A and Section B. Section A was designed to acquire information about the respondents such as type of organization, years of experience, and nature of work. Section B contained a total of 45 questions, each for assessing one waste cause. A five-point Likert scale was used to measure the impact of each cause on waste (1–Insignificant, 2–Low, 3–Moderate, 4–High, 5–Severe). The questionnaire was developed on the Google Forms platform and is ***attached as*** Appendix I. Data was acquired between 16 November 2023 and 23 December 2023 using online platforms and approaching different departments. Google Forms was distributed to the targeted respondents using WhatsApp professional groups and through emails. Project Managers (PM) working in highway construction firms in Pakistan were deemed appropriate as questionnaire survey respondents due to their relevant professional knowledge and experience. All the targeted respondents were Civil Engineers registered with the Pakistan Engineering Council (PEC). This study used convenience sampling, frequently used in construction management studies, to collect samples from the overall population.

Testing of reliability of questionnaire survey data through statistical analysis is deemed essential. Cronbach alpha (α) was used to check the internal reliability of the 5-Point Likert Scale questionnaire responses through Cronbach’s Alpha using Statistical Package SPSS^®^. Cronbach’s Alpha is computed through Equation 1.


$$\begin{array}{c} \alpha =(N/N-1)[1-(\sum \;\sigma_{x}^{2}/\sigma_{y}^{2})] \end{array}$$ (1)

“α” is Cronbach Alpha (0 < α < 1),“N” is Number of items, “*σ*_*x*_^*2*^” is Variance, “*σ*_*y*_^*2*^” Sum of variances. Table 5 ***shows Cronbach’s alpha values for internal consistency*** [43].

**Table 5:** Values of Cronbach Alpha for Reliability.

Reliability	Excellent	Good	Acceptable	Questionable	Poor	Unacceptable
Α	0.9 & Above	0.8 - 0.9	0.7 - 0.8	0.6 - 0.7	0.5 - 0.6	0.5 & Below

### 3.3. Relative importance of causes

Relative Importance Index (RII) helps to statistically sort all causes as per the index score; the topmost factors are deemed as the most significant waste-contributing causes [12,24].


$$\begin{array}{c} \text{RII}=\;\sum \text{W}/\left(\text{A}\times \text{N}\right) \end{array}$$ (2)

“W” is the weightage assigned to each cause, “A” is the largest value of weightage, and “N” is the number of respondents. RII value lies between 0 and 1(0 ≤ RII ≤ 1). RII of each cause gives the overall ranking of each cause. The greater the value of RII the more its significance is. **Table 6** ***shows RII values for High, Medium, Medium, and low-importance levels*** [44].

**Table 6:** Values of RII for Importance Level.

Importance Level	High	High-Medium	Medium	Low-Medium	Low
**RII Values (0 ≤ RII ≤ 1)**	0.8 & Above	0.6 - 0.8	0.4 - 0.6	0.2 - 0.4	0 - 0.2

### 3.4. Statistical analysis

Kruskal–Wallis Test is a non-parametric test for evaluating the significant difference in perceptions between various respondent groups [9,12]. Statistical Package SPSS^®^ was used with a confidence interval of 95% and a significance level of 5%. The null hypothesis implies that the medians of categories are statistically equal and will be rejected for a p-value less than 0.05.

Spearman’s rho intercorrelation matrix is used to examine the level of correlation between various variables. The value of the Correlation Coefficient rho determines the level of correlation, if the rho > 0.5 the correlation is high, if the value of rho is between 0.3–0.5 the correlation is moderate [45]. The correlation analysis was performed using Statistical Package SPSS^®^. If the majority of the correlations are found significant at the 0.01 level (two-sided); 99% confidence level, FA can be performed on the data set to determine future direction [9].

Principal Component Analysis (PCA) is a type of exploratory FA, that generates a reduced structure of factor groups that represent relationships among the variables [46]. The analysis groups the latent variables (waste causes in this study) into identical and similar clusters/components through the analysis of variances [47]. The adequacy of data for suitability of factor analysis is checked through Kaiser–Meyer–Olkin (KMO) test and the Bartlett sphericity test which can be performed using Statistical Software SPSS^®^ [46,47]. The result of the KMO test ranges between 0 and 1. Studies suggest that KMO values above 0.8 show adequacy of data while values between 0.6–0.79 are satisfactory and below 0.60 are inadequate [46]. Bartlett’s Test of Sphericity tests the relationship among variables for suitability for a structure, it should be significant (p-value < 0.05) for FA to be conducted appropriately [9]. In FA, the values of each variable are computed to assess their retention. Variables with Eigenvalues of greater than 1 are retained for analysis [47]. Many construction management studies have used FA [9,48,49].

## 4. Analysis of results

PMs from shortlisted highway construction firms were approached through email and WhatsApp groups. The response rate of the questionnaire survey was 42% as 132 responses were received out of 300 questionnaires circulated. 127 Valid responses were considered for the study because the remaining 5 responses were declared invalid because of being considerably incomplete. Similar studies have been conducted with sample sizes between 100 and 150 [15,17,23] (*see sample sizes in* Table 1 *above*). All the respondents are currently working in prominent construction firms in Pakistan as PMs, with 51% participation from contractor firms, 26% from client firms, and 23% from consultants. The vast majority (55%) of respondents were from public firms, 36% from private firms, and 9% remaining from semi-public firms. Participation from senior professionals with 16 years and the above experience was maximum (54%), this was followed by participation from mid-level professionals with 6–15 years of experience (21%) and the least participation from junior professionals with 5 years and less experience (9%) ***(Respondents profile is shown in*** Table 7***).***

**Table 7:** Respondents Profile - Project Managers (PM).

Property	Item	Frequency	Percentage
Nature of Experience	Client	34	26
	Contractor	65	51
	Consultant	29	23
Type of Firm	Public	70	55
	Private	46	36
	Semipublic	12	9
Years of Experience	Senior 16 &above	69	54
	Mid 6–15	40	21
	Young 0–5	19	15

The overall Cronbach Alpha for 45 variables computed by Statistical Package SPSS^®^ was 0.949. This shows that excellent reliability (α > 0.9). The examination of values for “Cronbach Alpha if item deleted” reveals that no value is greater than 0.949, meaning that deletion of any variable does not increase the value of overall Cronbach Alpha. This allows us to retain all variables for subsequent analysis ***(values “Cronbach Alpha if item deleted” attached as*** Appendix II***).***

Out of the total of 45 causes included in the study, 40 causes fell in the importance level of Medium-High (RII > 0.6) and the remaining 5 causes fell in the Medium importance level (0.4 < RII < 0.6), while there was no cause with which can be classified in Low importance level (RII < 0.4). Top five most significant causes were mistakes of surveyors (HWC24), faulty drawings (HWC3), incompetence of quantity surveyors (QS) (HWC23), Faulty work requiring rework (HWC38), and poor worker’s skills (HWC21). While ordering errors (HWC7), complex design (HWC1), delays in delivery of materials (HWC10), suppliers’ errors (HWC8), and occurrence of accidents (HWC44) were the least significant among the 45 evaluated causes. Out of the most significant HWCs, waste mistakes of surveyors (HWC24) and incompetence of QSs (HWC38), have been introduced by the expert panel of this study remaining all have been adapted from the literature. ***The ranking of HCW as per RII is attached as*** Appendix III**.**

The sample was divided into three sub-groups according to organizational type (Sub-group A), experience (Sub-group B), and nature of work of respondents (Sub-group C). Sub-group A was classified according to public, private, and semi-public organizations; Sub-group B was classified according to junior, mid-level, and senior professionals; while Sub-group C was classified into client, contractor, and consultant organizations. Kruskal Wallis Test was applied using Statistical Package SPSS^®^ to see significant differences in the perceptions of respondents within Groups A, B, and C. The result of sub-group A reveals that all p-values are greater than 0.05 meaning that there is no significant difference in the respondent’s perceptions between the three categories for all causes. However, for the sub-group-B, all p-values less HWC 23 (incompetence of QS) and HWC 29 (lower than the designed level of subgrade) were more than 0.05. This shows that these two factors were impacted by the perceptions of young, mid-level, and senior professionals. Likewise, the result of Sub-group C also reveals that p-values of all causes less HWC 21(poor workers skills) and HWC 42 (Site Spread over Very Long Length) were greater than 0.05 which reveals that these two causes were affected by the perceptions of respondents working with contractor, client, and consultant firms. It is important to note that the p-value for HWC 21(poor worker’s skills) is only 0.009 which is substantially lower than 0.05 and was the cause most affected by the respondents’ perceptions. Worker’s skill is the prime responsibility of the contractor while the other two categories are involved in the performance audit of the contractor, therefore this cause is perceived entirely differently by the three categories within the group. ***Kruskal Wallis Test results are attached as*** Appendix IV.

The level of correlation between the HWC was determined through Spearman’s rho correlation matrix (***attached as*** Appendix V). Most of the correlations were found significant at 95% (*) and 99% (**) confidence intervals (two-tailed). Therefore, FA can be performed on the entire data set [11,45].

FA was applied to the responses on 45 HWCs to reduce the large number of variables and identify a small number of coherent clusters of these variables. KMO test and the Bartlett sphericity test were applied to verify data adequacy. KMO for 45 items was computed as 0.872 which is excellent for performing the FA. Bartlett’s test of sphericity was 3126.594 with a significance level of 0 ***(See*** Table 8 ***for results).*** Consequently, the null hypothesis was rejected, implying that the correlation matrix was not an identity matrix. Both parameters used for adequacy of data support applying PCA. Moreover. Eigenvalues of all variables were computed higher than 1 which allowed us to retain all 45 variables for analysis. Subsequently, the 45 items were correlated with the PCA and then rotated using the Varimax method to generate a reduced structure. PCA was re-run to extract only the meaningful variables with factor loadings greater than 0.5. After the final run reduced structure, of 25 variables clustered in 7 groups, was generated. The 7 factors groups included in the reduced structure were able to explain 66.770% of the total variance which fulfills the construct validity criterion being greater than 60% [9,48,49]. ***(See*** Table 9 ***for FA for Total Variances)***

**Table 8:** KMO and Bartlett’s Test Results.

Kaiser-Meyer-Olkin Measure of Sampling Adequacy.		0.872
Bartlett’s Test of Sphericity	Approx. Chi-Square	3126.594
	Df	990
	Sig.	0.000

**Table 9:** FA for Total Variances.

Component	Initial Eigenvalues			Rotation Sums of Squared Loadings		
	Total	% of Variance	Cumulative %	Total	% of Variance	Cumulative %
1	7.739	30.955	30.955	3.369	13.478	13.478
2	2.157	8.628	39.583	3.079	12.317	25.795
3	1.678	6.711	46.294	2.404	9.616	35.410
4	1.613	6.452	52.746	2.082	8.327	43.738
5	1.308	5.233	57.979	2.050	8.199	51.936
6	1.136	4.544	62.523	1.913	7.650	59.586
7	1.062	4.247	66.770	1.796	7.184	66.770

The Cronbach Alpha of the 25 items (HWC) included in the reduced structure was 0.902, which shows the data’s excellent reliability (Table 10 shows the ***Reduced structure)***.

**Table 10:** Reduced Structure - HWFs.

Highway Waste Causes	Highway Waste Factors	Factor Loadings	Cronbach Alpha	Highway Waste Causes	Highway Waste Factors	Factor Loadings	Cronbach Alpha
HWC1	HWF 1 Design	0.775	0.763	HWC25	HWF 4 Workers	0.653	0.808
HWC2		0.716		HWC26		0.689	
HWC3		0.748		HWC27		0.699	
HWC5		0.687		HWC33		0.637	
HWC11	HWF 2 Storage	0.769	0.681	HWC42		0.646	
HWC12		0.737		HWC34	HWF 5 Waste Management	0.819	0.655
	HWC13		0.692		HWC35		0.634
HWC19	HWF 3 Survey	0.733	0.844	HWC39	HWF 6 Site Management	0.798	0.726
HWC20		0.697		HWC40		0.741	
HWC23		0.728		HWC41		0.599	
HWC24		0.742		HWC43	HWF 7 External	0.799	0.764
	HWC29		0.639		HWC44		0.812
				HWC45	0.561		

## 5. Findings and discussion

Out of seven HWFs evaluated by PCA, 6 factors were supported by at least 3 causes with factor loading more than 0.50, which is a criterion for retaining a factor in FA. However, Waste Management (HWF5) was retained despite being supported by only two causes with factor loadings more than 0.50 because of being essentially important. Although this practice has been discouraged, FA studies in construction management have retained factors with 2 variables as well [47,48]. Details of seven HWFs computed in this study are as under:

### 5.1. HWF1: Design

This factor group comprises of 4 causes, including Complex Design (HWC1), Frequent Changes (HWC2), Faulty Drawings(HWC3), and Less Planning Time(HWC5). All these causes are related to highway construction’s design/planning stage. Factor loadings of all variables supporting HWF1 are greater than 0.60 which is considered good for retaining the factor group. The overall Cronbach alpha is greater than 0.70 which is also considered acceptable for the reliability of its data. This factor also meets the criteria of being supported by four factors with loadings greater than 0.40 [46,47]. In most previous studies, design-related CW causes/factors have been given due recognition (*See* Table 2 *above*). In the context of highway projects, frequent design changes has been evaluated as one of the most significant causes of wastage in road projects [15,16].

### 5.2. HWF2: Storage

This factor group comprises 3 HWCs, including bulk procurement in advance (HWC11), lack of storage space(HWC12), and Multiple Storage Spaces spread along long stretches (HWC13). All these causes are related to the storage of materials. Factor loadings of all variables computed for HWF -2 are also greater than 0.60, which is considered good for retaining the factor. The overall Cronbach alpha of the group is greater than 0.60 which is considered questionable for the reliability of its data, however, it does not fall in poor/unacceptable limits. This factor also meets the minimum criteria of being supported by three items[46,47]. Storage of materials has been evaluated as an important factor cluster in many other CW studies (See Table 2 above). In the context of highway projects, poor material segregation and poor storage arrangements have been included in the list of causes of ABC wastage [15].

### 5.3. HWF3: Survey

This factor group consists of 5 HWCs which include Non-availability of Appropriate Surveying Equipment (HWC19), Faulty/Malfunctioning of Equipment (HWC20), Incompetence of QSs (HWC23), Mistakes of Surveyors (HWC24), and Lower than Designed Level (HWC29),. The factor group represents causes that result in survey-related flaws because of workers’ incompetence and equipment inadequacies. Factor loadings of all variables computed for HWF -3 are also greater than 0.60 which is considered good for its inclusion. The overall Cronbach alpha of the group is greater than 0.80 which is considered good for the data reliability. This factor also meets the criteria of being supported by four items with more than 0.40 factor loading [46,47]. The result of RII computed in this study evaluates the Mistake of Surveyors (HWC24), and the Incompetency of QSs (HWC38), among top three causes of waste in highway construction. Although, Survey related factor has not been discussed in previous studies, the causes contributing to this factor were assigned to other factors (See Tables 1–2 above). In the previous studies, the mistakes and incompetency of workers-related waste causes have been discussed in worker/ Human Resource related factors, whereas nonavailability and malfunctioning of survey equipment were grouped with Equipment cluster (See Tables 1–2 above). In a recent study on causes of Aggregate Base Course wastage, QSs mistakes and improper use of equipment have been ranked at eighth and ninth place respectively out of 23 items as per RII score [15]. In another study on infrastructure projects including inter alia road construction, the cause of waste associated with inexperienced workers has been grouped in execution factor groups while equipment-related causes of waste have not even been included in the analysis [14]. A study on causes of waste in highway construction conducted in 2008 ignored equipment-related cause/factor [16]. The introduction of this new factor examined in this study emphasizes the importance of the role of land surveying as well as quantity surveying in highway construction projects.

### 5.4. HWF4: Workers

This factor group has 5 causes of wastage, consisting of Fast Pace of Work (HWC25), Problems with Attitude and Behaviour of Workers (HWC26), Lack of Awareness of Wastage (HWC27), Lack of Coordination amongst stakeholders (HWC33), and Site Spread over Long Length (HWC42). Most of the causes are related to the workers. Factor loadings of all variables computed for HWF4 are also greater than 0.60, which is considered good for retaining the factor. The overall Cronbach alpha of the group is greater than 0.80, which is considered good for the reliability of its data. This factor qualifies the criteria of being supported by a minimum of four items with greater than 0.40 loading [46,47]. As per the previous studies, two factors (Mistakes of Surveyors and Incompetency of QSs) have relevance with the Worker Factor group, which have been grouped with the Survey factor in this study based on the computation of FA. Worker-related factors appear to have more applicability on waste in building construction vis-a-vis highway & infrastructure projects as evident from the outcome of previous studies (***See*** Tables 1 ***&*** 2 ***above****).*

### 5.5. HWF5: Waste management

This factor group contains only two causes, including Absence of a Waste Management Plan (HWC34) and the Use of Wrong Construction Methods (HWC35). These causes are pertaining to waste management. Both causes are related to waste management. Factor loadings of all variables computed for HWF5 are also greater than 0.60 which are factors considered good for retaining the factor. The overall Cronbach alpha of the group is greater than 0.60 which is considered questionable, however do not fall in poor/unacceptable limits for the data reliability. This factor fails to qualify the criteria of being supported by a minimum of three items [46,47]. However, due to the immense importance of waste management in the literature, this factor has been retained. Many similar studies have concluded that a lack of waste management plan is among the most significant causes of CW [9,25]. The RII-based ranking of Use of Wrong Construction Methods and absence of CWM Plan computed in this study are sixth and seventeenth respectively. Both waste causes were also examined in a study on the waste of ABC in highway projects [15].

### 5.6. HWF6: Site management

This factor group contains 3 causes Unsuitable Site (HWC39), Site Restricting Equipment Operation (HWC40), and Remote Site/ Wilderness (HWC41). These causes are related to the site management. Factor loadings of two variables computed are also greater than 0.50 which are deemed acceptable for retaining the factor. The overall Cronbach alpha of the group is greater than 0.60 which is questionable but not in the poor/unacceptable limits for the reliability of its data. This factor qualifies the criteria of being supported by three items [46,47]. The literature review reveals that site management is one of the most frequently analyzed waste factors (See Table 2 above), however, the associated waste causes have not been placed among the most significant causes by most of the previous studies (See Table 1 above). The RII conducted in this study shows that none of the waste causes pertaining to the site are amongst the top ten significant causes.

### 5.7. HWF7: External

There are three causes in this group including Theft and Vandalism incidents (HWC43), Occurrence of Accidents (HWC44), and Bad Weather Conditions (HWC45). These causes are related to external factors. Factor loadings of three variables are greater than 0.50 which are considered acceptable for retaining the factor. The overall Cronbach alpha of the group is greater than 0.70 which is within acceptable limits for the reliability of its data. This factor qualifies the criteria of being supported by three items [46,47]. Previous studies have examined the external factors frequently (See Table 2). In the context of highway projects, Extreme weather has been evaluated as one of the most significant causes of waste generation [14,16]. RII computed in this study shows the relatively lower ranking of these causes *(See* Table 8 *above).*

### 5.8. Comparison with previous studies on CW

This study distinguishes itself by focusing on micro-level causes of construction waste (CW) in highway projects, whereas previous research primarily analyzed macro-level and more generalized waste causes. Specifically, this study introduces expert-identified, site-specific causes of highway CW, including those related to workers and site operations (see Table 1). For instance, while Naji et al. (2022) and Nazech et al. (2008) examined broader CW causes in highway and infrastructure projects in Qatar and Indonesia [14,16], and Perera et al. (2022) focused on waste causes related to a single highway construction material in Sri Lanka [15], most prior research has concentrated on building projects, often identifying similar generic waste causes [21–29,42].

A key distinction of this study is that among the five most significant highway CW causes identified through RII analysis, two—Mistakes of Surveyors and Incompetence of Quantity Surveyors—are being introduced for the first time, while the remaining three—Faulty Drawings, Rework, and Poor Worker Skills—have been previously recognized as significant (see Table 1).

Moreover, the factor analysis highlights a novel contribution: this study identifies a new waste causative factor related to “Survey Work,” comprising five specific micro-level waste causes. Despite the critical role of survey work in highway construction, previous studies have not examined it as a distinct waste-contributing factor (see Table 2). While six other waste factors analyzed in this study have been explored in prior research, this study delves deeper into the micro-level causes that contribute to each macro-level factor, offering a more granular understanding of highway CW generation. Each factor assessed in this research is supported by waste causes specific to highway construction, reinforcing the micro-level nature of the study

### 5.9. Suggested CWM strategies

Many studies have formulated CWM strategies based on the causative factors of waste generation. This study proposes waste management strategies from the literature corresponding to the analyzed factors. Table 11 ***shows proposed waste management strategies corresponding to each waste factor***.

**Table 11:** Proposed CWM Strategies.

Waste Group	Potential CWM Strategies adapted from the literature
HWF 1 Design	• Design strategy training for capacity building of the project design team. • Integrated Project Design for flexibility and adaptability at the design stage. • Use of Design and Build contract for reducing design issues between the contractor and designer. • Automation including application of BIM for better CW management.
HWF 2 Storage	• Appropriate storage arrangement for minimizing waste. • Ordering Just in Time(JIT) to facilitate material flow schedule. • Mechanical handling to prevent wastage of materials. • Maintenance of proper documentation by recording and measuring CW streams.
HWF 3 Survey	• Hiring of skilled and experienced staff for operating sophisticated survey equipment and software. • Specialized training of surveyors and QSs for using equipment properly. • Careful handling of tools and equipment on site. • Allocation of required equipment in operational condition.
HWF 4 Workers	• Waste minimization culture within the institution • On-site supervision and training sessions for employees on optimal utilization of the materials. • Use of skilled and experienced labour and supervisors. • Proper site control and supervision • Employing designated waste managers to oversee waste management.
HWF 5 Waste Management	• Plan 3 R Waste Management Strategy for reduction, reuse, and recycling of waste. • Proper onsite administration of 5 Ms (men, material, money, machines, and management) • Coordination between all stakeholders during all phases of construction projects. • Implement automated construction waste management. • Carry out feasibility studies for waste estimation.
HWF 6 Site	• Provision of security, security lighting, and temporary fencing at the site. • Proper reconnaissance of alignment of the project. • Use of proper earthwork machinery.
HWF 7 External	• Adopt effective and flexible planning techniques to reduce the waste resulting from bad weather • Provision of security, security lighting, and temporary fencing at the site • Waste management plan to be formulated in consonance with Health and safety plan, Traffic Management Plan and Environmental Impact Analysis.

## 6. Conclusion

Highway construction projects are distinct from other construction projects because of their peculiar set of management and logistics challenges. The consumption of enormous quantities of construction materials and susceptibility to wastage necessitate the formulation of waste management strategies specific to highway projects. While the literature confirms the depth and breadth of research on construction waste in building projects it reports scarcity in the realm of highway and infrastructure projects. The study questions the applicability of generic waste-impacting factors on highway construction projects and introduces new relevant causative factors. The subsequent evaluation through the Relative Importance Index (RII) and Factor Analysis (FA) reveals considerable singularity and distinctiveness of waste in highway construction projects. The most significant causes of highway construction waste evaluated through RII are: (1) mistakes of surveyors, (2) faulty drawings, (3) incompetence of QSs, (4) faulty/substandard work, and (5) poor workers’ skills. This study establishes seven causative factors of highway waste through factor analysis: (1) design, (2) storage, (3) survey, (4) workers, (5) waste management, (6) site management, and (7) external. Subsequently, waste management strategies corresponding to each factor are suggested from previous studies.

This study contributes to the existing body of knowledge by introducing waste causative factors specific to highway construction projects, which will serve as a foundation for further research on waste management for highway projects. The study will assist highway construction professionals in handling waste more effectively and efficiently. This study has been conducted in the context of the construction industry of Pakistan, similar to most of the other waste management studies that are carried out in the auspices of a particular region/country. The findings of this study are likely to have considerable generalizability in developing countries in the same region due to similar conditions. Future research on construction waste in highway projects in different parts of the world should validate the findings of this study. This study has suggested general waste management strategies from the literature addressing each waste causative factor evaluated through Factor Analysis. Sequel to this study, detailed research should be conducted on the waste management strategies for highway construction projects similar to the research conducted on building projects [21–23]. Perera et al. (2021) has conducted research on wastage of Aggregate Base Course one of the main constituents of the highway project [15]. Future research may also explore the causes of the wastage of other construction materials like sub-base, sub-grade, asphalt and aggregates used in highway construction. Material wastage in highway projects can significantly impact project cost and time performance. Researchers can investigate the relationship between cost overruns/delays and wastage.

## 7. Limitations of the study

This research identified general waste management strategies from the literature without employing a rigorous research analysis approach, as its primary focus was on identifying and evaluating waste causes. A more detailed study is needed to develop a comprehensive framework for waste management strategies that specifically address the highway waste causative factors identified in this study. Moreover, the findings of this study should be validated through multiple case studies to ensure their applicability across different highway construction projects. This approach would also facilitate the refinement of waste mitigation measures, ensuring they are tailored to specific project contexts and effectively address the micro-level causes of construction waste.

## Supporting information

Appendices 1 Questionnaire Survey – Waste Causes in Highway Projects. (DOCX)

Appendices 2 Cronbach Alpha if Item Deleted. (DOCX)

Appendices 3 Ranking of HWC as per RII. (DOCX)

Appendices 4 Result of Kruskal Wallis Test. (DOCX)

Appendices 5 Spearman’s rho Correlation Matrix. (DOCX)
