# Supplementary material for: Exploring the critical waste factors affecting highway construction projects in Pakistan
Source: PLoS One. 2025 May 28;20(5):e0323841. doi: 10.1371/journal.pone.0323841 (PMC12119017; doi:10.1371/journal.pone.0323841)
Supplement: Appendices 1 — (DOCX) [file pone.0323841.s001.docx]

**Appendix I**

| **HWC** | **Highway Construction Cause** | **Impact of Each HWC on Wate Generation**  **Rating as per Likert Scale (1 – 5)**  **1 is Insignificant and 5 is Severe** | | | | |
| --- | --- | --- | --- | --- | --- | --- |
| HWC1 | Complex Design | 1 | 2 | 3 | 4 | 5 |
| HWC2 | Frequent Changes | 1 | 2 | 3 | 4 | 5 |
| HWC3 | Faulty Drawings | 1 | 2 | 3 | 4 | 5 |
| HWC4 | Delay in Drawings Distribution | 1 | 2 | 3 | 4 | 5 |
| HWC5 | Less Planning Time | 1 | 2 | 3 | 4 | 5 |
| HWC6 | Taking Off Errors (over or underestimation) | 1 | 2 | 3 | 4 | 5 |
| HWC7 | Ordering Errors | 1 | 2 | 3 | 4 | 5 |
| HWC8 | Suppliers Errors | 1 | 2 | 3 | 4 | 5 |
| HWC9 | Poor Quality and Wrong Specifications | 1 | 2 | 3 | 4 | 5 |
| HWC10 | Delay in Delivery of Materials from suppliers | 1 | 2 | 3 | 4 | 5 |
| HWC11 | Bulk Procurement in Advance | 1 | 2 | 3 | 4 | 5 |
| HWC12 | Lack of Storage Space | 1 | 2 | 3 | 4 | 5 |
| HWC13 | Multiple Storage Spaces spread along long stretch of road alignment | 1 | 2 | 3 | 4 | 5 |
| HWC14 | Distance of Work Site from storage places | 1 | 2 | 3 | 4 | 5 |
| HWC15 | Inappropriate/Inadequate Storage Arrangements | 1 | 2 | 3 | 4 | 5 |
| HWC16 | Mishandling by Equipment during Transportation to site | 1 | 2 | 3 | 4 | 5 |
| HWC17 | Mishandling of Material during Loading and Unloading | 1 | 2 | 3 | 4 | 5 |
| HWC18 | Non-availability of Appropriate Earthmoving Machines & Equipment | 1 | 2 | 3 | 4 | 5 |
| HWC19 | Non-availability of Appropriate Surveying Equipment | 1 | 2 | 3 | 4 | 5 |
| HWC20 | Faulty/Malfunctioning of Equipment | 1 | 2 | 3 | 4 | 5 |
| HWC21 | Poor Workers Skills | 1 | 2 | 3 | 4 | 5 |
| HWC22 | Poor Capacity of Designers | 1 | 2 | 3 | 4 | 5 |
| HWC23 | Incompetence of Quantity Surveyors | 1 | 2 | 3 | 4 | 5 |
| HWC24 | Mistakes of Surveyors | 1 | 2 | 3 | 4 | 5 |
| HWC25 | Fast Pace of Work | 1 | 2 | 3 | 4 | 5 |
| HWC26 | Problems with Attitude and Behavior of Workers | 1 | 2 | 3 | 4 | 5 |
| HWC27 | Lack of Awareness of Wastage | 1 | 2 | 3 | 4 | 5 |
| HWC28 | How Poor Working Conditions for workers | 1 | 2 | 3 | 4 | 5 |
| HWC29 | Lower than the Designed Level of the Subgrade Layer | 1 | 2 | 3 | 4 | 5 |
| HWC30 | Poor Supervision of Work | 1 | 2 | 3 | 4 | 5 |
| HWC31 | prolonged Halting of Work by Consultant / Client | 1 | 2 | 3 | 4 | 5 |
| HWC32 | Halts between Different Processes due to Engineering Practices | 1 | 2 | 3 | 4 | 5 |
| HWC33 | Lack of Coordination amongst stakeholders | 1 | 2 | 3 | 4 | 5 |
| HWC34 | absence of Waste Management Plan | 1 | 2 | 3 | 4 | 5 |
| HWC35 | Use of Wrong Construction Methods | 1 | 2 | 3 | 4 | 5 |
| HWC36 | Frequent Movement of Materials from one site to another | 1 | 2 | 3 | 4 | 5 |
| HWC37 | Material Segregation and Sorting issues | 1 | 2 | 3 | 4 | 5 |
| HWC38 | Faulty/Substandard Work (Requiring Rework) | 1 | 2 | 3 | 4 | 5 |
| HWC39 | Unsuitable Site (Rough/rocky/marshy/undulating) | 1 | 2 | 3 | 4 | 5 |
| HWC40 | Site Restricting Equipment Operation | 1 | 2 | 3 | 4 | 5 |
| HWC41 | Remote Site / Wilderness (accessibility issues) | 1 | 2 | 3 | 4 | 5 |
| HWC42 | Site Spread over Very Long Length impacts | 1 | 2 | 3 | 4 | 5 |
| HWC43 | Theft and Vandalism incidents | 1 | 2 | 3 | 4 | 5 |
| HWC44 | Occurrence of Accidents | 1 | 2 | 3 | 4 | 5 |
| HWC45 | Bad Weather Conditions | 1 | 2 | 3 | 4 | 5 |

**QUESTIONNAIRE SURVEY – WASTE CAUSES IN HIGHWAY PROJECTS**

This survey will be used to identify and evaluate waste causes in highway projects based on the responses of highway construction professionals. You are required to rate impact of each cause on waste generation in highway construction projects from 1 to 5 (1 is insignificant and 5 is severe). Participation in this survey is entirely voluntary and will mean your explicit consent. You are not required to share your personal information. Data acquired from this survey will only be used for academic purposes and will not be transferred/communicated to any individual/organization in any case.
