# Supplementary material for: Exploring the critical waste factors affecting highway construction projects in Pakistan
Source: PLoS One. 2025 May 28;20(5):e0323841. doi: 10.1371/journal.pone.0323841 (PMC12119017; doi:10.1371/journal.pone.0323841)
Supplement: Appendices 2 — (DOCX) [file pone.0323841.s002.docx]

**Appendix II**

**Cronbach Alpha if Item Deleted**

| ID | Scale Mean if Item Deleted | Scale Variance if Item Deleted | Corrected Item-Total Correlation | Cronbach's Alpha if Item Deleted |
| --- | --- | --- | --- | --- |
| HWC1 | 146.13 | 540.478 | 0.334 | 0.949 |
| HWC2 | 145.66 | 539.141 | 0.352 | 0.949 |
| HWC3 | 145.35 | 536.812 | 0.421 | 0.949 |
| HWC4 | 145.98 | 532.433 | 0.480 | 0.948 |
| HWC5 | 145.76 | 535.083 | 0.410 | 0.949 |
| HWC6 | 145.98 | 533.590 | 0.541 | 0.948 |
| HWC7 | 146.12 | 533.947 | 0.529 | 0.948 |
| HWC8 | 146.34 | 536.382 | 0.439 | 0.949 |
| HWC9 | 145.67 | 532.159 | 0.437 | 0.949 |
| HWC10 | 146.32 | 531.810 | 0.482 | 0.948 |
| HWC11 | 145.95 | 539.107 | 0.321 | 0.949 |
| HWC12 | 145.80 | 531.927 | 0.494 | 0.948 |
| HWC13 | 145.96 | 533.928 | 0.474 | 0.948 |
| HWC14 | 146.01 | 531.268 | 0.535 | 0.948 |
| HWC15 | 145.63 | 529.037 | 0.586 | 0.948 |
| HWC16 | 146.05 | 525.737 | 0.647 | 0.947 |
| HWC17 | 145.95 | 530.140 | 0.570 | 0.948 |
| HWC18 | 145.84 | 529.493 | 0.595 | 0.948 |
| HWC19 | 145.64 | 527.665 | 0.551 | 0.948 |
| HWC20 | 145.85 | 527.009 | 0.579 | 0.948 |
| HWC21 | 145.42 | 526.561 | 0.652 | 0.947 |
| HWC22 | 145.60 | 526.730 | 0.623 | 0.948 |
| HWC23 | 145.37 | 526.029 | 0.637 | 0.947 |
| HWC24 | 145.34 | 526.745 | 0.657 | 0.947 |
| HWC25 | 145.88 | 530.236 | 0.479 | 0.948 |
| HWC26 | 145.80 | 527.360 | 0.635 | 0.947 |
| HWC27 | 145.45 | 527.887 | 0.611 | 0.948 |
| HWC28 | 145.70 | 528.009 | 0.602 | 0.948 |
| HWC29 | 145.57 | 528.971 | 0.507 | 0.948 |
| HWC30 | 145.50 | 525.606 | 0.654 | 0.947 |
| HWC31 | 145.55 | 528.281 | 0.526 | 0.948 |
| HWC32 | 146.03 | 529.432 | 0.539 | 0.948 |
| HWC33 | 145.62 | 528.033 | 0.604 | 0.948 |
| HWC34 | 145.68 | 535.385 | 0.422 | 0.949 |
| HWC35 | 145.44 | 533.854 | 0.550 | 0.948 |
| HWC36 | 145.58 | 528.183 | 0.589 | 0.948 |
| HWC37 | 145.95 | 530.234 | 0.579 | 0.948 |
| HWC38 | 145.40 | 528.809 | 0.595 | 0.948 |
| HWC39 | 145.80 | 535.927 | 0.445 | 0.949 |
| HWC40 | 145.97 | 533.479 | 0.514 | 0.948 |
| HWC41 | 145.77 | 530.303 | 0.544 | 0.948 |
| HWC42 | 145.87 | 532.935 | 0.510 | 0.948 |
| HWC43 | 146.07 | 526.570 | 0.545 | 0.948 |
| HWC44 | 146.38 | 529.073 | 0.548 | 0.948 |
| HWC45 | 145.77 | 531.137 | 0.483 | 0.948 |
