# Supplementary material for: Exploring the critical waste factors affecting highway construction projects in Pakistan
Source: PLoS One. 2025 May 28;20(5):e0323841. doi: 10.1371/journal.pone.0323841 (PMC12119017; doi:10.1371/journal.pone.0323841)
Supplement: Appendices 3 — (DOCX) [file pone.0323841.s003.docx]

**Appendix III**

**Ranking of HWC as per RII**

| **ID** | **Cause** | **RII** | **Ranking** |
| --- | --- | --- | --- |
| HWC24 | Mistakes of Surveyors | 0.753 | 1 |
| HWC3 | Faulty Drawings | 0.750 | 2 |
| HWC23 | Incompetence of Quantity Surveyors | 0.746 | 3 |
| HWC38 | Faulty/Substandard Work (Requiring Rework) | 0.742 | 4 |
| HWC21 | Poor Workers Skills | 0.737 | 5 |
| HWC35 | Use of Wrong Construction Methods | 0.734 | 6 |
| HWC27 | Lack of Awareness of Wastage | 0.731 | 7 |
| HWC30 | Poor Supervision of Work | 0.721 | 8 |
| HWC31 | Prolonged halting of Work by Consultant / Client | 0.712 | 9 |
| HWC29 | Lower than the Designed Level of the Subgrade Layer | 0.707 | 10 |
| HWC36 | Frequent Movement of Materials from one site to another / Double-handling | 0.706 | 11 |
| HWC22 | Poor Capacity of Designers | 0.699 | 12 |
| HWC33 | Lack of Coordination among stakeholders | 0.698 | 13 |
| HWC15 | Inappropriate/Inadequate Storage Arrangements | 0.694 | 14 |
| HWC19 | Non-availability of Appropriate Surveying Equipment | 0.693 | 15 |
| HWC2 | Frequent Changes | 0.687 | 16 |
| HWC9 | Poor Quality and Wrong Specifications | 0.687 | 16 |
| HWC34 | Absence of Waste Management Plan | 0.685 | 18 |
| HWC28 | Poor Working Conditions for Workers | 0.680 | 19 |
| HWC5 | Less Planning Time | 0.666 | 20 |
| HWC45 | Bad Weather Conditions | 0.666 | 20 |
| HWC41 | Remote Site / Wilderness (accessibility issues) | 0.663 | 22 |
| HWC39 | Unsuitable Site (Rough/ rocky/ marshy/undulating) | 0.661 | 23 |
| HWC12 | Lack of Storage Space | 0.661 | 23 |
| HWC26 | Problems with Attitude and Behaviour of Workers | 0.660 | 25 |
| HWC18 | Non-availability of Appropriate Earthmoving Equipment | 0.652 | 26 |
| HWC20 | Faulty/Malfunctioning of Equipment | 0.649 | 27 |
| HWC42 | Site Spread over Very Long Length | 0.647 | 28 |
| HWC25 | Fast Pace of Work | 0.644 | 29 |
| HWC11 | Bulk Procurement in Advance | 0.631 | 30 |
| HWC37 | Material Segregation and Sorting issues | 0.630 | 31 |
| HWC13 | Multiple Storage Spaces spread along long stretch of road alignment | 0.628 | 32 |
| HWC17 | Mishandling of Material during Loading/ Unloading | 0.628 | 32 |
| HWC40 | Site Restricting Equipment Operation | 0.625 | 34 |
| HWC4 | Delay in Drawings Distribution | 0.624 | 35 |
| HWC6 | Taking Off Errors (over or underestimation) | 0.624 | 36 |
| HWC14 | Distance of Work Site from storage places | 0.617 | 37 |
| HWC32 | Halts between Different Processes due to Engineering Practices | 0.614 | 38 |
| HWC16 | Mishandling by Equipment during Transportation | 0.606 | 39 |
| HWC43 | Theft and Vandalism incidents | 0.603 | 40 |
| HWC7 | Ordering Errors | 0.595 | 41 |
| HWC1 | Complex Design | 0.592 | 42 |
| HWC10 | Delay in Delivery of Materials from suppliers | 0.556 | 43 |
| HWC8 | Suppliers Errors | 0.553 | 44 |
| HWC44 | Occurrence of Accidents | 0.540 | 45 |
