# Supplementary material for: Exploring the critical waste factors affecting highway construction projects in Pakistan
Source: PLoS One. 2025 May 28;20(5):e0323841. doi: 10.1371/journal.pone.0323841 (PMC12119017; doi:10.1371/journal.pone.0323841)
Supplement: Appendices 4 — (DOCX) [file pone.0323841.s004.docx]

**Appendix IV**

**Result of Kruskal Wallis Test**

| **ID** | **Organizational Type**  **Sub-group A** | **Experience**  **Sub-group B** | **Nature of Work**  **Sub-group C** |
| --- | --- | --- | --- |
| HWC1 | 0.849 | 0.630 | 0.375 |
| HWC2 | 0.829 | 0.601 | 0.371 |
| HWC3 | 0.638 | 0.867 | 0.274 |
| HWC4 | 0.017 | 0.760 | 0.072 |
| HWC5 | 0.076 | 0.769 | 0.024 |
| HWC6 | 0.828 | 0.066 | 0.131 |
| HWC7 | 0.817 | 0.427 | 0.138 |
| HWC8 | 0.926 | 0.304 | 0.587 |
| HWC9 | 0.840 | 0.302 | 0.608 |
| HWC10 | 0.749 | 0.508 | 0.400 |
| HWC11 | 0.530 | 0.849 | 0.455 |
| HWC12 | 0.716 | 0.408 | 0.355 |
| HWC13 | 0.986 | 0.660 | 0.393 |
| HWC14 | 0.455 | 0.982 | 0.211 |
| HWC15 | 0.452 | 0.900 | 0.184 |
| HWC16 | 0.830 | 0.635 | 0.144 |
| HWC17 | 0.799 | 0.156 | 0.149 |
| HWC18 | 0.864 | 0.079 | 0.640 |
| HWC19 | 0.220 | 0.150 | 0.495 |
| HWC20 | 0.369 | 0.209 | 0.682 |
| HWC21 | 0.641 | 0.526 | 0.009 |
| HWC22 | 0.432 | 0.185 | 0.215 |
| HWC23 | 0.463 | 0.043 | 0.129 |
| HWC24 | 0.910 | 0.214 | 0.157 |
| HWC25 | 0.381 | 0.859 | 0.572 |
| HWC26 | 0.376 | 0.673 | 0.726 |
| HWC27 | 0.848 | 0.350 | 0.177 |
| HWC28 | 0.461 | 0.851 | 0.615 |
| HWC29 | 0.841 | 0.038 | 0.471 |
| HWC30 | 0.928 | 0.237 | 0.231 |
| HWC31 | 0.588 | 0.121 | 0.211 |
| HWC32 | 0.945 | 0.627 | 0.605 |
| HWC33 | 0.638 | 0.915 | 0.544 |
| HWC34 | 0.833 | 0.773 | 0.573 |
| HWC35 | 0.862 | 0.159 | 0.230 |
| HWC36 | 0.843 | 0.291 | 0.313 |
| HWC37 | 0.296 | 0.456 | 0.255 |
| HWC38 | 0.932 | 0.397 | 0.230 |
| HWC39 | 0.224 | 0.601 | 0.213 |
| HWC40 | 0.838 | 0.430 | 0.556 |
| HWC41 | 0.636 | 0.682 | 0.764 |
| HWC42 | 0.971 | 0.514 | 0.049 |
| HWC43 | 0.972 | 0.050 | 0.868 |
| HWC44 | 0.656 | 0.775 | 0.722 |
| HWC45 | 0.857 | 0.209 | 0.245 |
