# Supplementary figures and images for: Exploring the critical waste factors affecting highway construction projects in Pakistan
Source: PLoS One. 2025 May 28;20(5):e0323841. doi: 10.1371/journal.pone.0323841 (PMC12119017; doi:10.1371/journal.pone.0323841)

**Appendix V**

**Spearman’s rho Correlation Matrix**
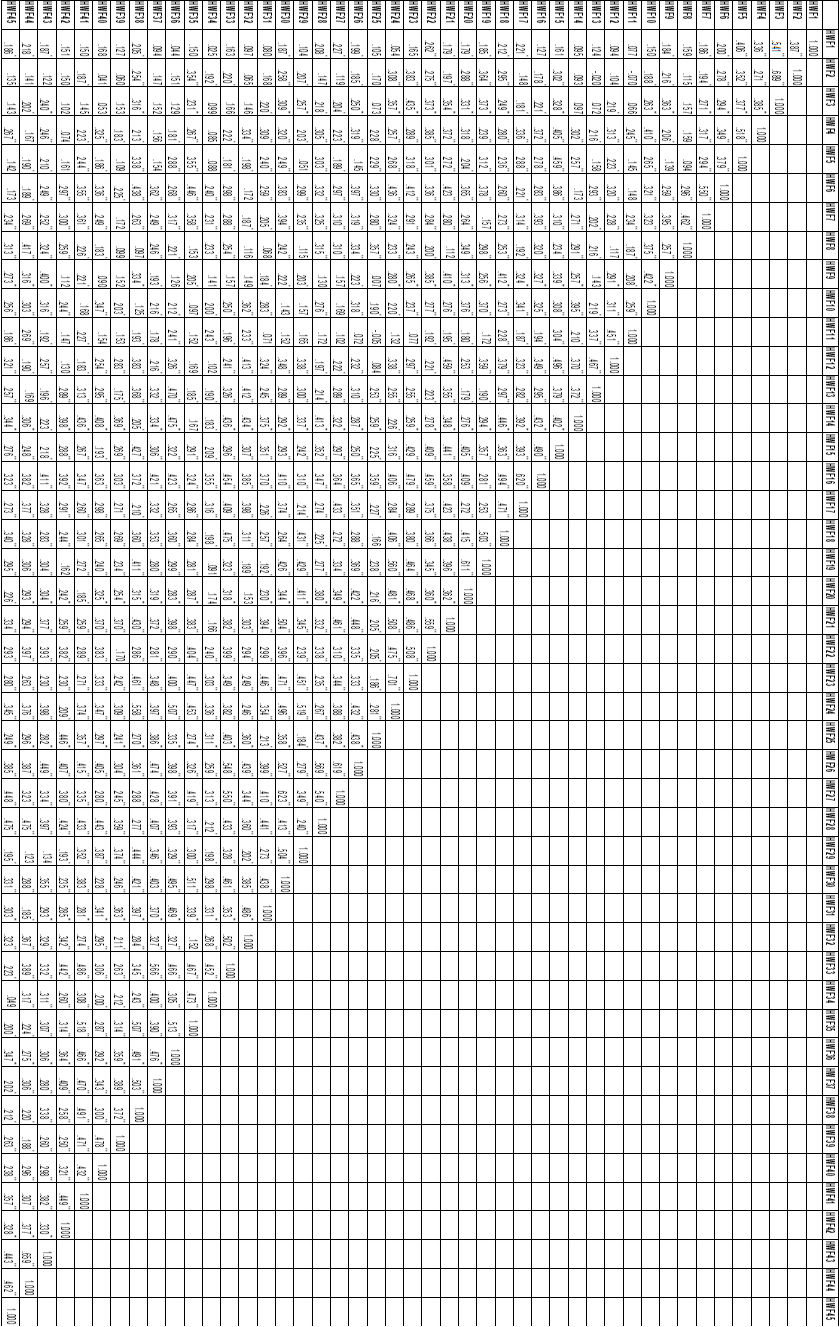

Supplement: Appendices 5 — (DOCX) [file pone.0323841.s005.docx]
